# Predictors of caregiver support intervention outcomes for refugees in Jordan: a three-path mediational study

**DOI:** 10.1186/s13034-025-00995-1

**Published:** 2025-12-07

**Authors:** M. J. D. Jordans, G. V. Koppenol-Gonzalez, H. Al Hmoud, A. F. Guevara, F. Steen, H. Mansour, B. A. Kohrt

**Affiliations:** 1 Research and Development Department, War Child Alliance, Helmholtzstraat 61G, 1098 LE Amsterdam, The Netherlands; 2 Amsterdam Institute for Social Science Research, University of Amsterdam, Amsterdam, The Netherlands; 3 Program Department, War Child Alliance, Amman, Jordan; 4 Center for Global Mental Health Equity, George Washington University, Washington, DC USA

**Keywords:** Quality of care, Common and specific factors, Predictors of outcome, Parenting, Low and middle income countries

## Abstract

**Background:**

More evidence is needed for understanding how evidence-based psychological interventions work, especially when implemented by briefly trained non-professionals in low- and middle-income settings. The nine-session caregiver support intervention (CSI) is such an evidence-based intervention, aiming to improve parental wellbeing and parenting skills. Specifically, this study aims to assess if and how a set of quality of care indicators explain intervention outcomes amongst participants from refugee settlements in Jordan.

**Methods:**

We conducted a three-path mediational model, wherein levels of facilitators’ competency (using the ENhancing Assessment of Common Therapeutic factors tool [ENACT]), facilitators’ fidelity of implementing CSI, caregivers’ attendance, and caregivers’ adoption of the key intervention skills and strategies (i.e. mechanisms of action) are predictive of intervention outcomes. Outcome measures include the Warwick-Edinburgh Mental Wellbeing Scale to assess caregiver wellbeing, Kessler Psychological Distress measure to assess caregivers’ psychological distress symptoms, Brief Parenting Questionnaire to assess warm and responsive parenting and harsh parenting and Kid(dy)-KINDL for Parents to assess children’s psychosocial wellbeing.

**Results:**

The study, amongst caregivers (*n* = 588) and facilitators (*n* = 51), demonstrates that baseline to endline intervention improvements in parenting, caregiver wellbeing, and caregiver-reported child wellbeing are all (fully or partly) mediated by a pathway of higher levels of facilitator competencies leading to higher levels of participants’ attendance. Higher attendance in turn leads to higher levels of adoption of key intervention strategies by participants. Higher adoption in turn leads to the bespoke positive outcomes (indirect effects: 0.50 [SE = 0.14]; 0.90 [SE = 0.26]; 0.92 [SE = 0.29], respectively). This pathway does not play out for caregiver distress as an outcome (−0.20 [SE = 0.13]).

**Conclusion:**

Both common factors (foundational therapeutic competencies among the service providers) and specific factors (active ingredients of the intervention) are relevant in predicting outcomes following CSI—albeit through different pathways. This study provides support for using competency assessments, attendance tracking, and monitoring participant’s skill adoption as a framework for the evaluation and improvement of quality of care.

**Supplementary Information:**

The online version contains supplementary material available at 10.1186/s13034-025-00995-1.

## Background

In response to a large need for mental health care in low-resource settings, the last decades have seen new interventions being developed that follow a task-sharing approach, which entails that mental health care is delivered by briefly-trained facilitators that do not have a formal mental health training. There is increasing evidence for the effectiveness of such interventions implemented in low- and middle-income countries (LMIC) [1, 2]. Albeit trailing somewhat, we see the evidence base for child- and family-focused interventions also emerging [3, 4]. One of these interventions is the caregiver support intervention (CSI). CSI consists of nine group sessions and has a dual aim to improve the wellbeing of caregivers and promote positive parenting practices—in order to have a downstream effect on improving the wellbeing of children in adversity [5].

With the availability of effective interventions and the confidence that the task-shifted model of intervention delivery is feasible, the research agenda should increasingly shift towards creating a better understanding of what aspects of the intervention or intervention delivery are driving participant benefits [6]. In brief, this can mean investigations related to *how* the evidence-based intervention is best delivered (i.e. implementation science) or *what* mechanisms of interventions are driving positive outcomes (i.e. unpacking the black box of psychological interventions). This understanding is important in order to improve quality, potency and subsequently cost-effectiveness of interventions. This is especially salient when aiming to scale up services in resource-scarce settings.

Understanding what makes psychological interventions work has been a central question in psychotherapy research for decades [7]. Central to this debate is whether common factors or specific factors are the main contributors to outcomes. Specific factors refer to techniques that are specific to an intervention and as they are described in manuals [8]. Specific factors are also described as ‘mechanisms of action’ or ‘active ingredients.’ For example, a recent review synthesized the evidence for 27 active ingredients in preventing or treating youth anxiety and depression [9]. This can be assessed through fidelity checklists or instruments designed to capture change in mechanisms of action [10]. Common factors refer to factors that all interventions have in common (such as a therapeutic alliance, empathy, expectations) [11, 12]. The notion that all psychotherapies are equivalent because of common factors that are shared in all treatments was coined as the Dodo bird verdict in 1936, with a study comparing 17 meta-analyses often cited to support this position [13]. One way this gets translated into research and practice is through the assessment of therapist competencies [14], which has been the rationale behind the EQUIP platform (https://equipcompetency.org/en-gb) that makes competency assessment tools available to ensure helpers provide safe, high quality psychosocial support and mental health care [15, 16].

Decades of research has resulted in studies showing the clear benefits of common factors and especially the therapeutic relationship [12, 17, 18], in studies demonstrating evidence for specific factors or active ingredients [19–21], in studies demonstrating the relevance of both [8], as well as meta-analytic studies pointing towards the absence of evidence for either common and specific factors [7]. While that in itself calls for more research into empirically validated working mechanisms, this is especially the case when it comes to psychological interventions implemented by non-specialists in LMIC where such research is still scarce. The current study aims to address that gap and is implemented against the background of this debate. Following results from a randomized controlled trial of CSI in Lebanon [5, 22], the present study aims to investigate what specific and common factors are predictive of intervention outcomes. Specifically, we were interested to assess if and how a set of quality of care indicators (competency, attendance, fidelity and mechanisms of action) explain participants benefiting from CSI [6].

## Methods

### Setting

This study took place in multiple refugee settlements and governates in Jordan. Specifically, Zaatari camp—War Child Center, Emirati-Jordanian Camp (EJC), Mafraq governorate, Zarqa governorate, and Amman governorate—with mixed populations including Syrian, Palestinian and Iraqi refugees, as well as host community. Across these settings different organizations were responsible for implementing the caregiver support intervention, namely War Child, Qudrat Association for Community Development, Al Kiram, Al Oun, and Sanabel Al Khair.

### Design

Aiming to understand whether levels of facilitators’ competency (i.e. common factors), facilitators’ fidelity of implementing CSI (i.e. specific factors), caregivers’ attendance (i.e. dosage), and caregivers’ adoption of the key intervention strategies or mechanisms of action (i.e. specific factors) are predictive of intervention outcomes, we conducted a three-path mediational model, wherein two mediators intervene in a series between an independent and a dependent variable. The serial mediation model combines data from the CSI facilitators (i.e. competency, fidelity) and CSI participants (i.e. attendance, mechanisms of action, outcomes).

### Sample

First, following a purposive sampling strategy, 51 facilitators participated in the study and were trained to implement CSI. The facilitators were recruited from ongoing programs being implemented by the organizations mentioned above, selecting facilitators that had: (1) no prior experience in implementing psychosocial or mental health interventions; (2) 1–3 years of experience, and; (3) over 3 years of experience in implementing psychosocial or mental health interventions. This range was included to reduce the risk of a ceiling effect in the reporting of the facilitator-level predictor variables. All facilitators were over 18 years of age. Second, the study involved 588 caregivers receiving CSI to evaluate change from pre- to post-intervention. We recruited caregivers of children in the 4–12-year age range that were residents in the above-mentioned settlements and that provided consent to participate in the study. Caregivers below 18 years of age were excluded. To evaluate downstream outcomes of CSI on (non-participating) children of the (participating) caregivers, we asked each of the caregivers to report on one of their children (index child between 4 and 12; in case of multiple children the index child was selected at random). Third, 181 caregivers’ children (aged 7–12; same child as the aforementioned index child) were also included in the before and after assessments to evaluate child-reported downstream effects.

### Instruments

#### Caregiver outcomes

The Warwick-Edinburgh Mental Wellbeing Scale (WEMWBS) was used to assess caregiver wellbeing [23]. The WEMWBS is a 14-item instrument (total score range 14–70), which was used as the co-primary outcome for the CSI trial [22]. The internal consistency of the WEMWBS in this study was good (*α* = 0.858, 95% CI = 0.400–0.874). The Kessler Psychological Distress measure (K10) was used to assess caregivers’ psychological distress symptoms [24]. It is commonly used in research in LMICs, including our prior CSI studies in the Middle East. In the current study the K10 showed good internal consistency (*α* = 0.899, 95% CI = 0.887–0.910). We administered the Brief Parenting Questionnaire (BPQ), a 24-item self-report measure for use with parents of children ages 3–12, to assess warm and responsive parenting and harsh parenting. The instrument was developed specifically for the evaluation of CSI among Syrian refugees [25]. The internal consistency of the BPQ was good (*α* = 0.861, 95% CI = 0.844–0.876). Children’s psychosocial wellbeing was measured using the Kid-KINDL for Parents for children aged 7–12 and the Kiddy-KINDL for Parents for children 3–6 [26].

#### Child outcomes

The 24-item KINDL for Children (7–12 years of age) was used to assess child-reported quality of life and wellbeing, demonstrating acceptable internal consistency (*α* = 0.768, 95% CI = 0.712–0.815). This instrument was used to assess the hypothesis that the participation of caregivers in CSI leads to downstream changes at the level of the caregivers’ children. The child-level outcome was not included in the mediation model, but was included to demonstrate relevance for the primary outcomes for this study (i.e. caregiver-level outcomes) given CSI’s ultimate aim to have a downstream effect on children’s wellbeing.

#### Indicators

The hypothesized indicators can be separated by facilitator-level variables (competency and fidelity) and participant-level variables (attendance and mechanisms of action). Therapeutic competency of facilitators was measured with the 13-item ENACT tool that assesses common factors in psychological treatments using observations. It is scored at four levels per competency (level 1 = potentially harmful, level 2 = no sufficient competency, level 3 = basic competency, level 4 = mastery) and two types of attributes (helpful, harmful) [15], wherein the attributes are descriptions of example observable behavior for each of the four levels. Intervention fidelity, the degree to which a program is delivered as intended by the developers [27], was assessed using the CSI fidelity tool, consisting of 12–21 items per session, answer options 0–2 (not done, partially done, fully done) (see Additional file 4). Attendance was registered for each participant for each of the nine CSI session (absent, present), using a session log that is completed by the facilitators. The CSI Skills Use instruments was developed (specifically for this intervention) to assess the usage of skills and strategies that are being promoted during the intervention, operationalizing the intervention’s mechanisms of action. The CSI Skills Use scale includes 5 items (with response options 1–5), including items such as ‘use of stress-management skills’, ‘use of emotional regulation skills’, and ‘use of positive attention’—each with examples that link it to the intervention content (see Additional file 5).

### Intervention

CSI is a nine-session group intervention, with weekly sessions to 10–12 caregivers (separate for men and women). CSI is facilitated by two trained non-mental health professionals (native Arabic speaker, from the geographic locations of implementation, minimum age 24 years) that receive an 8-day training. The intervention has a dual focus on strengthening caregiver wellbeing and strengthening parenting skills. This is based on robust evidence that (1) persistently high levels of parental stress (as is common in conflict situations) is associated with caregivers engaging in increased harsh parenting and decreased supportive or nurturing interactions, and (2) that compromised parenting impacts children’s mental health and psychosocial wellbeing [5]. Sessions 1–4 are focused on understanding and managing stress, disengaging from “thinking too much”, and coping with anger and frustration. Sessions 5–8 focus on increasing awareness of the impact of stress on parenting, positive parenting strategies and use of non-violent discipline methods. Session 9 involves a review and closing of the intervention. In all but the final session, participants learn a new relaxation skills.

### Procedures

All outcome measures and the CSI Skills Use assessment were administered through interviews by a group of research assistants (*n* = 8) that had received a 4-day training in communication and interviewing skills, research ethics and procedures. Outcome assessments were conducted immediately before and after participating in CSI. Fidelity assessments were done through self-reporting by the facilitators after completing each sessions, and through observations of 10% of the sessions by the CSI supervisors. Competency assessments of all facilitators (*n* = 51) were done before and after the training (using standardized role-plays), as well as at midterm (using observations of sessions) during the CSI cycle by raters trained in using ENACT on the EQUIP platform. Supervision was offered to all facilitators on a bi-weekly basis and provided by supervisors with extensive experience in implementing CSI.

### Analyses

First, descriptive analyses were conducted on the sample demographic characteristics at baseline, on each of the outcomes at baseline and endline, and on each of the indicators at their respective time points (ENACT pre-training, post-training and in-session; fidelity and attendance across all sessions; and Skills Use at baseline and endline). Second, changes in outcomes and in Skills Use from baseline to endline were analyzed with paired sample *t* tests correcting for multiple testing (Bonferroni correction *α* = 0.007). Facilitator competencies (ENACT) were also analyzed for changes over time from pre- to post-training to in-session by testing the differences in counts of the level 1, level 2, level 3 and level 4 scores with a Friedman test and Conover post-hoc comparisons with Bonferroni correction for multiple testing. The competency scores were also analyzed using the counts of the attribute scores (harmful, helpful). For further analyses concerning competencies in relation to other variables, we used the competency scores post-training, as these are most in line with the intended use of the competency assessment.

After analyzing the descriptives and changes over time, the following steps were taken to examine the relationship between the indicators and the outcomes: (1) fidelity was analyzed by comparing the facilitator self-reports and the co-facilitator observations with the supervisor observations to determine which was the best indicator; (2) the intra-class correlations (ICC) reflecting clustering of caregivers within facilitator groups were examined per outcome to determine whether to aggregate or disaggregate the data before continuing with the next steps; (3) the correlations (Spearman’s rho) between indicators and outcomes were analyzed to examine the one-on-one relationships between them, and additionally; (4) we checked for possible suppressor and moderator variables by combining indicators and adding demographic variables in a linear regression model for each outcome.

Based on the results per step, the following decisions were made: (1) exclude fidelity scores due to ceiling effects and restriction of range and use Skills Use as a proxy for fidelity as they are conceptually similar, albeit at participant rather than facilitator level; (2) disaggregate the facilitator data by linking facilitator scores to individual caregiver scores; (3) based on the correlation results, i.e., ad-hoc, we first defined conceptual mediation models before actually testing the different path coefficients in order to examine combinations of indicators (at facilitator and caregiver level) in relation to the outcomes. Also, distress was examined as a moderator to explain unexpected negative correlations. The details of these decisions are given in the “Results” section below. We conducted a serial mediation with two mediators using the PROCESS macro for R [28] and the other analyses were conducted in JASP [29].

## Results

Table 1 presents socio-demographic details for the caregivers and the facilitators. The average age of the caregivers was 35.9 (SD = 8.1), for the index child 8.2 (SD = 2.5) and for child respondents 9.2 (SD = 2.1). Both caregiver and facilitator samples are heavily skewed towards female participants, while the child sample is more equally divided by gender.

**Table 1:** Sample characteristics at baseline

		Caregivers (%) (*n* = 588)	Facilitators (%) (*n* = 51)	Index child^a^ (%) (*n* = 588)	Children^b^ (%) (*n* = 181)
Gender	Female	534 (91.1)	46 (90.2)	282 (48)	84 (46.4)
	Male	54 (8.9)	4 (7.8)	303 (51.5)	97 (53.4)
	Missing		1 (2)	3 (0.5)	
Nationality	Syrian	463 (78.8)	16 (31.4)		
	Jordanian	119 (20.2)	32 (62.7)		
	Other (Lebanese, Iraqi or Palestinian)	6 (1.0)	3 (5.9)		
Highest educational level	No schooling	33 (5.6)	0		
	Primary	159 (27)	0		
	Secondary	189 (32.1)	0		
	High school	129 (22)	11 (21.6)		
	Vocational	32 (5.5)	7 (13.7)		
	University/Bachelor	46 (7.8)	23 (45.1)		
	University/Master	0	4 (7.8)		
	Other	0	6 (11.8)		
Marital status	Single	5 (0.8)			
	Married	536 (91.2)			
	Widowed	20 (3.4)			
	Divorced	15 (2.5)			
	Separated	8 (1.4)			
	Missing	4 (0.7)			
Working status	Yes	167 (28.4)			
	No	415 (70.6)			
	Missing	6 (1)			
Type of housing	Tented settlement	5 (0.8)			
	Apartment	222 (37.8)			
	House	201 (34.2)			
	Caravan	157 (26.7)			
	Other	3 (0.5)			
Financial perception	Very financially secure	6 (1)			
	Somewhat financially secure	69 (11.8)			
	In some financial trouble	226 (38.4)			
	In deep financial trouble	287 (48.8)			
Relationship index child	Mother	511 (86.9)			
	Father	50 (8.5)			
	Grandmother	12 (2)			
	Grandfather	1 (0.2)			
	Other relative	12 (2)			
	Non-relative guardian	2 (0.4)			

^a^The index child is the child the caregiver thought of when filling in the surveys

^b^Children included as respondents

Baseline to endline comparisons demonstrated statistically significant improvement on all the outcome measures. The smallest change was seen on psychological distress (*d* = 0.33, 95% CI 0.25–0.42) and the largest change was seen on parenting (*d* = 0.58, 95% CI 0.49–0.67). Moreover, children’s reports also show a significant improvement in psychosocial wellbeing over time (*d* = 0.34, 95% CI 0.18–0.50). See Table 2 for details.

**Table 2:** Baseline and endline outcomes and comparisons

	T1 Mean (SD)	T2 Mean (SD)	*t*	*df*	*p*	Cohen’s *d* (95% CI)
*Caregivers*						
K10 Total	29.305 (9.347)	26.33 (8.964)	−8.098	587	<0.001	0.33 (0.25–0.42)
Parenting	52.793 (6.528)	55.978 (5.25)	14.136	587	<0.001	0.58 (0.49–0.67)
Parental warmth and responsiveness	36.26 (4.623)	38.147 (3.823)	11.215	587	<0.001	0.46 (0.38–0.55)
Harsh parenting	11.467 (2.727)	10.17 (2.336)	−12.061	587	<0.001	0.50 (0.41–0.58)
KINDL Total	93.771 (13.605)	99.393 (13.014)	9.995	587	<0.001	0.41 (0.33–0.49)
WEMBWS Total	48.502 (9.894)	52.712 (9.749)	10.17	587	<0.001	0.42 (0.34–0.50)
*Children*						
KINDL Total	94.418 (0.885)	98.302 (0.956)	4.397	164	<0.001	0.34 (0.18–0.50)

K10 = Kessler 10

Overall, fidelity was very high (at least 97% done across all sessions, taking partially and fully done together, only fully done was still at least 95%), also when reported by a co-facilitator (at least 97% done) or by the supervisor (at least 94% done). There was too little variance in fidelity to use in further analysis. On average 6 of the 9 sessions (66.0%) were attended by the caregivers, with a higher average for female participants (68.1%) compared to male (45.8%), as well as a higher attendance in the first four sessions (69.8%) compared to the second part of the intervention (61.1%). The total score in Mechanism of Action changed from 16.65 (SD = 3.647) to 19.29 (SD = 3.01), a significant difference (*t*(587) = 14.784, *p* < 0.001; *d* = 0.61, 95% CI = 0.52–0.70).

Table 3 presents the competency outcome results. Overall, we see statistically significant changes over time and in the expected direction (i.e. less frequent scoring levels 1 and 2 and more frequent scoring levels 3 and 4 over time). This change was observed mainly from pre- to post-training, and from pre-training to in-session observations. The same trend was observed for the individual attributes that make up the scoring for level 1 (harmful) and levels 3 and 4 (helpful).

**Table 3:** Facilitator competency scores over time (*n* = 51), Friedman tests and Conover post-hoc comparisons

		Mean	SD	*χ*^2^ (*df*) overall	*t* (*df*) pre	*t* (*df*) post
Level 1: Potentially harmful	Pre-training	1.902	0.831	73.34 (2)***		
	Post-training	0.980	0.678		4.456 (100)***	
	In-session	0.196	0.401		8.581 (100)***	4.126 (100)***
Level 2: No sufficient competency	Pre-training	8.961	2.425	25.45 (2)***		
	Post-training	6.451	2.508		4.494 (100)***	
	In-session	6.353	3.045		4.238 (100)***	0.255 (100)
Level 3: Basic competency	Pre-training	3.863	2.592	18.39 (2)***		
	Post-training	5.824	2.114		4.284 (100)***	
	In-session	4.569	2.193		1.910 (100)	2.374 (100)
Level 4: Mastery	Pre-training	0.275	0.723	60.47 (2)***		
	Post-training	1.451	1.616		4.304 (100)***	
	In-session	3.294	2.468		7.780 (100)***	3.476 (100)**
Harmful attributes	Pre-training	1.922	0.845	71.43 (2)***		
	Post-training	1.020	0.761		4.236 (100)***	
	In-session	0.196	0.401		8.471 (100)***	4.236 (100)***
Helpful attributes	Pre-training	18.333	7.453	33.03 (2)***		
	Post-training	24.863	7.370		4.651 (100)***	
	In-session	26.706	9.890		5.251 (100)***	0.600 (100)

* *p* < 0.05, ** *p* < 0.01, *** *p* < 0.001, *p* values are corrected for multiple testing (Bonferroni)

Before analyzing the relationships between indicators and outcomes, we checked the ICCs of caregivers within intervention groups (facilitator-level). Given the small ICCs on all the outcomes (around 0.05), except for caregiver-reported child wellbeing (around 0.10) and the decrease in ICC from baseline to endline, we considered it unlikely that there was clustering at the facilitator level and disaggregated the data, linking the facilitator scores to each caregiver outcome score. Additional file 1 shows the partial correlations for all the indicators with the outcomes, conditioned on baseline scores. All the correlations between attendance and the outcomes were low, but significant and positive with parenting, parental warmth and responsiveness, and caregiver wellbeing. When looking at possible suppressors (Additional file 2), the other indicators did not change the relationship between attendance and the outcomes. The correlations between competencies and the outcomes were very low. When looking at possible suppressors, attendance seemed to slightly increase the correlations with distress and wellbeing and therefore was interpreted as a possible suppressor variable. There was a significant negative correlation between competencies and caregiver-reported child wellbeing. All the correlations between Skills Use and the outcomes were significant (negative with harsh parenting and distress, and positive with all the other outcomes). Finally, attendance had significant positive correlations with Skills Use (*ρ* = 0.222) and competencies had a significant positive correlation with attendance (*ρ* = 0.304).

In terms of moderators (caregiver age, gender, nationality, educational level, perception of financial situation, and total number of persons in the household), most of the models including them did not explain significantly more variance compared to the null model with only the indicator and outcome at baseline (Additional file 3). The only exception was attendance showing a significant interaction effect with gender on distress (*b* = 0.096, SE = 0.030, *p* = 0.002). For competencies (levels and attributes) none of the models including the moderators explained more variance, although some spurious interaction effects were found (e.g. with financial perception, educational level, household). After closer inspection the interaction was due to very small categories of the demographics and was therefore not considered meaningful.

**Fig. 1:**
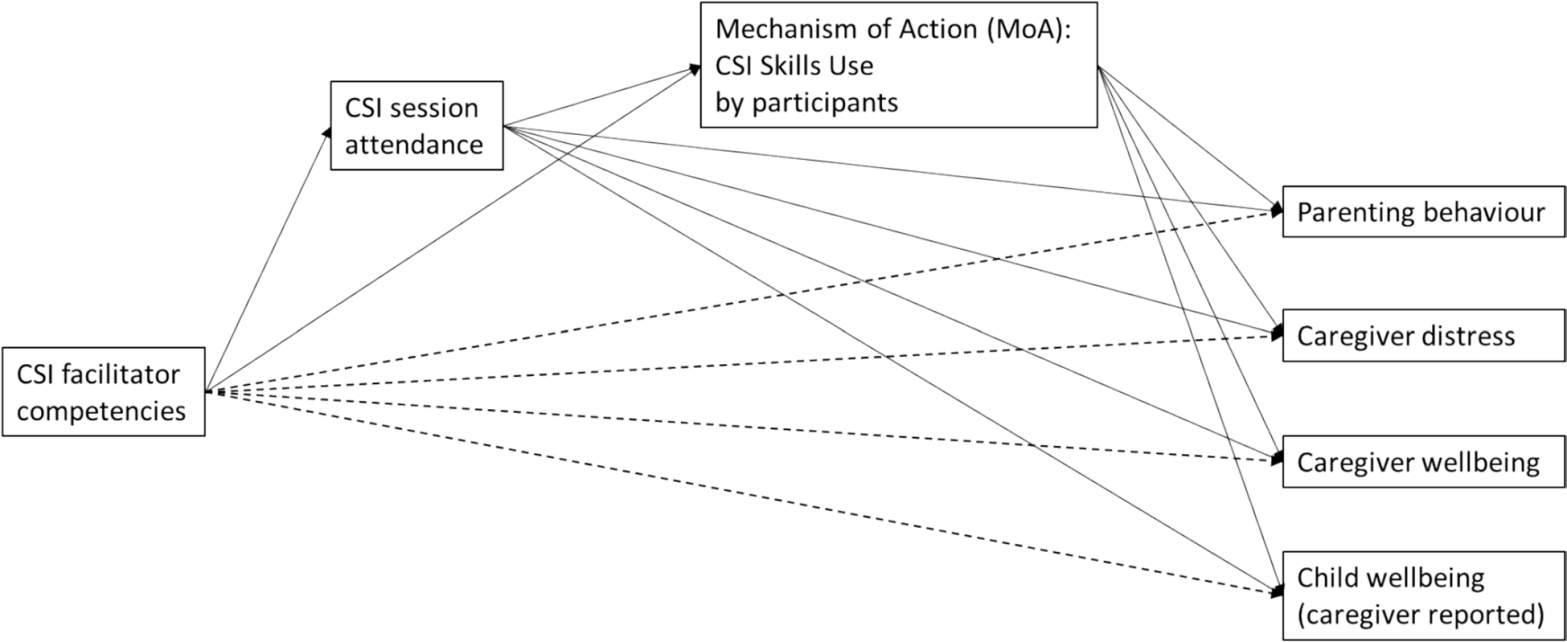
Conceptual model. CSI = caregiver support intervention; all four outcomes present in the model are pre-to-post change scores

Following the abovementioned results, we arrived at a multiple mediation model that consisted of competencies as the independent variable, CSI outcomes as the dependent variables (parenting, parental distress, parental wellbeing and parent-reported child wellbeing) and attendance and ‘mechanisms of action’ as hypothesized mediators. See Fig. 1 for the conceptual model being tested, consisting of a pathway from level of competency to the level of attendance, to change in mechanisms of action, to change in outcomes.

**Fig. 2:**
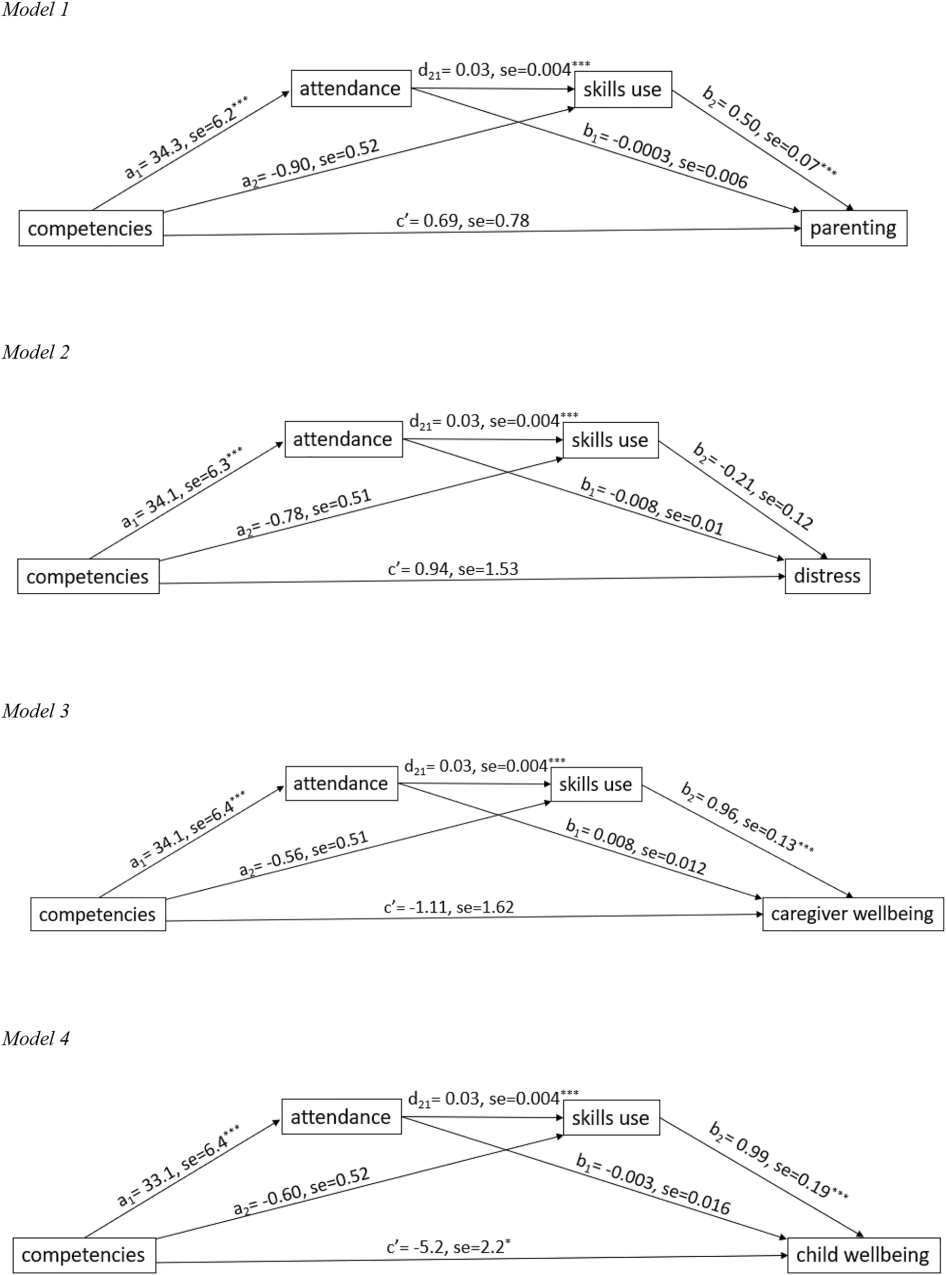
Serial mediation models

Below, we present the multiple mediation results for each of the four outcomes at endline, corrected for baseline scores: (i) parenting; (ii) caregiver distress; (iii) caregiver wellbeing, and; (iv) caregiver-reported child wellbeing. All results are presented in Fig. 2 (direct effects) and Table 4 (indirect and total effects), giving unstandardized estimates. First, (i) for parenting, the indirect effect of competencies through attendance and Skills Use is significant, as are the direct effects between competencies and attendance, attendance and Skills Use, and Skills Use and parenting. All the other effects are not significant. Second, (ii) for caregiver distress, only the direct effects between competencies and attendance, and attendance and Skills Use are significant. The results of post-hoc subgroup analyses distinguishing male and female caregivers show that the effect of attendance on distress is opposite for male and female caregivers (total indirect effects: female −0.73 [SE = 0.42]; male 3.61 [SE = 1.97]), explaining the non-significant results in the total sample. Third, (iii) for caregiver wellbeing, the indirect effect of competencies through attendance and Skills Use is significant, as are the direct effects between competencies and attendance, attendance and Skills Use, and Skills Use and caregiver wellbeing. All the other effects are not significant. Fourth, (iv) for caregiver-reported child wellbeing, the indirect effect of competencies through attendance and Skills Use is significant, as are the direct effects between competencies and attendance, attendance and Skills Use, and Skills Use and caregiver-reported child wellbeing. Also, the direct effect remains significant and is negative. This means that facilitator competencies seem to have a negative effect on caregiver-reported child wellbeing that is not explained by attendance nor Skills Use, given that the indirect and separate direct effects are all positive. So after accounting for the mediating effects of attendance and Skills Use, there remains a negative effect that is not explained by this model. Post-hoc analyses to investigate whether caregiver distress moderated the mediational pathway on caregiver-reported child wellbeing (i.e. between distress and competencies and between distress and attendance) showed that none of the interactions were significant and that adding distress to the model actually increased the negative direct effect from competencies to child wellbeing. So the negative relationship cannot be explained by adding distress as a moderator to the mediation model.

**Table 4:** Indirect and total effects (direct effects shown in Fig. 2 above)

	Predictor	Mediator 1	Mediator 2	Outcome	Indirect effect (SE)	Total indirect effect (SE)	Total effect (SE)
Model 1	Competencies	Attendance	n/a	Parenting	a_1_*b_1_ = −0.01 (0.22)		
	Competencies	n/a	Skills use	Parenting	a_2_*b_2_ = −0.45 (0.27)		
	Competencies	Attendance	Skills use	Parenting	a_1_*d_21_*b_2_ = 0.50 (0.14)***	0.04 (0.34)	0.73 (0.81)
Model 2	Competencies	Attendance	n/a	Distress	a_1_*b_1_ = −0.27 (0.38)		
	Competencies	n/a	Skills use	Distress	a_2_*b_2_ = 0.17 (0.16)		
	Competencies	Attendance	Skills use	Distress	a_1_*d_21_*b_2_ = −0.20 (0.13)	−0.31 (0.40)	0.63 (1.49)
Model 3	Competencies	Attendance	n/a	Caregiver wellbeing	a_1_*b_1_ = 0.27 (0.41)		
	Competencies	n/a	Skills use	Caregiver wellbeing	a_2_*b_2_ = −0.53 (0.50)		
	Competencies	Attendance	Skills use	Caregiver wellbeing	a_1_*d_21_*b_2_ = 0.90 (0.26)***	0.63 (0.66)	−0.48 (1.67)
Model 4	Competencies	Attendance	n/a	Child wellbeing	a_1_*b_1_ = −0.09 (0.54)		
	Competencies	n/a	Skills use	Child wellbeing	a_2_*b_2_ = −0.58 (0.54)		
	Competencies	Attendance	Skills use	Child wellbeing	a_1_*d_21_*b_2_ = 0.92 (0.29)**	0.24 (0.72)	−5.0 (2.2)*

SE = standard error, estimates are unstandardized

* *p* < 0.05, ** *p* < 0.01, *** *p* < 0.001

## Discussion

Over the past years much research has demonstrated that psychological interventions delivered by non-mental health professionals can be effective in LMIC [1, 3, 30, 31]. Much less is known about how these interventions work or what exactly contributes to the shown benefits. This study aimed to investigate whether predictors like facilitators’ competency, facilitators’ fidelity in implementing the intervention, participants’ attendance to the intervention, and participants’ usage of skills the intervention aims to promote (i.e. mechanisms of action) are associated with outcomes. The selection of indicators is based on a framework that outlines a sequence of measurable quality of care indicators to move towards delivering evidence-based practice at scale [6].

The main findings of this study are that before-to-after intervention improvements in caregiver wellbeing, caregiver-reported child wellbeing and parenting are all (fully or partly) mediated by a pathway of higher levels of facilitator competencies leading to higher levels of participants’ attendance. Higher attendance in turn leads to higher levels of adoption of key intervention strategies by participants. Higher adoption in turn leads to the bespoke positive outcomes. This pathway does not play out for caregiver distress as an outcome, which is not associated with the adoption of strategies. The reason for this might be that the intervention’s strategies are not sufficiently potent to address high-levels of distress—a hypothesis that is currently being researched. It is important to note that both attendance and intervention skills usage are significant direct predictors of outcomes, whereas this was not the case for competency. Competency not directly predicting outcomes has previously been reported in the literature [7, 32, 33]. This is maybe not so surprising, as one would expect facilitators’ therapeutic foundational skills to first impact participants’ perceptions and experience of the received support, which in turn contributes to positive changes. In this study that change occurs through influencing attendance.

### What does this mean?

First, at face value this means that the more competent the provider is (demonstrating higher levels foundational therapeutic skills), the more participants come back to the sessions (e.g. because they feel heard and understood by the facilitator), the more they pick up the skills and strategies that the intervention aims to instill (essentially a dose–response mechanism), which results in better outcomes (the key ingredients of the intervention have the intended effect).

Second, theoretically, going back to the common factors vs. specific factors debate summarized in the introduction, these findings essentially speak to the importance of both, rather than one over the other. Indeed, why would this be a zero-sum game? So, rather than the commonly investigated head-to-head comparisons trying to isolate the differential impact of common or specific factors in psychotherapy, it means moving beyond the dichotomy and investigating what combination of factors best predicts outcomes [8]. More recent studies have indeed demonstrated such interplay between common and specific factors—for example a combination of meditation minutes and social common factors contributing to mindfulness-based intervention outcomes [34]. The current study provides support for such a combined common and specific factors framework for predicting outcomes following a task-shifted psychological intervention in a LMIC. At the same time, it is important to note that we do not provide evidence for which specific factor or key intervention strategy, or which common factor, actually works—we only looked at a combination of merged common and merged specific factors. One can even wonder whether it is possible to evaluate isolated common or specific factors. In fact, a recent review by Cuijpers and colleagues show that no factor can be considered an empirically validated working mechanism [7].

Third, practically, the results speak to the relevance of routinely assessing a set of quality indicators that provides data that can be used to monitor the quality of implementation of an evidence-based intervention and can be used for data-driven quality improvement through supervision or further training [6]. Specifically, this study shows that competency changes over time, from pre- to post-training and further improving after practice, showing reduction in harmful behaviors to near absence—replicating earlier findings [35]. Especially post-training (pre-implementation) competency levels of facilitators are relevant, best predicting increased attendance and as being a feasible assessment point for future scaling. It also supports the relevance of improving common factor competencies during training, possibly through using pre-training competency levels to do so—a strategy that has already demonstrated to work in Lebanon [36]. Given the near-recent lack of a standardized approach to ensure competency assessment amongst non-specialized providers of psychological interventions in LMICs, the launch of the aforementioned EQUIP platform (being used in over 36 countries since 2022) providing competency assessment tools and resources for real-world practice is so relevant [16]. Kohrt and colleagues also demonstrate the assessment of change in competency scores from before training to after training to during supervision in seven LMICs (including the study in Lebanon mentioned above) using the EQUIP tools and platform.

As for attendance, this study shows the importance of keeping participants in the intervention (see also direct associations between attendance and outcomes). A recent study investigating the effects of intervention dose (through attendance) of a cognitive behavioral depression prevention intervention for adolescents showed that higher attendance predicted better outcomes (threshold of approximately 75% of the sessions attended) [37]. Higher levels of common factor competencies appear to be one way of manipulating this. In addition, from a quality-of-care perspective it is important to also use other strategies to reduce drop-outs. Reviews have shown that effective strategies exist to increase (mental) health treatment retention in high income settings [38], as well as in LMIC settings [39].

Although not designed or conceptualized as an indicator to routinely assess quality of care, given that the intervention skills usage (mechanism of action) score was the strongest individual predictor of outcomes and showed the largest before-to-after change, it merits considering routine use of a simple (5-item in this case) participant self-report instrument to gauge the level of uptake of key intervention strategies during the implementation process. As with competency and attendance levels, this allows supervisors to use data-driven or precision supervision techniques that are potentially more effective and less resource intensive than supervision that does not use such data. Using a simple instrument that operationalizes an intervention’s mechanism of change has also been used, and shown to mediate outcomes, for the WHO’s Group Problem Management Plus (gPM+) intervention [10].

Facilitators’ fidelity in implementing CSI was not included in the pathway models because of insufficient variance. High levels of fidelity are very common in global mental health interventions studies. This is probably unsurprising given that fidelity instruments are commonly developed for the purpose of assessment of adequate implementation in the context of a trial. Moreover, a review of specific intervention techniques has shown to not be predictive in symptom reduction [32]. For routine monitoring of quality of care, the existing, often long, very detailed fidelity measurement may therefore not be adequate and needs to be replaced by one that is shorter and predictive of outcomes.

There are a few limitations to be noted. The study combined data from facilitators and participants in one model, and aimed to have large samples of both populations, it was not powered for the mediation analyses. Furthermore, while the conceptual mediation model was defined before doing the analyses, the final model was not a priori defined. After several studies evaluating CSI [22, 40, 41], this is the first to showing downstream child-reported outcomes, yet the sample of enrolled children was too small to evaluate predictors of child-level outcomes and was therefore not included in the mediation model.

## Conclusion

Participants in a nine-session caregiver support intervention for refugee populations in Jordan report improvements on all outcomes. Before and after intervention changes on improved parenting, increased caregiver wellbeing and caregiver-reported children’s wellbeing are all mediated through a pathway from higher levels of competency to increased attendance to increased adoption of intervention strategies.

This study provides insight in the relevance of both common factors (foundational therapeutic competencies among the service providers) and specific factors (active ingredients of the intervention)—albeit through different pathways, wherein the former has an indirect contribution to outcomes and the latter a direct on. Moreover, in much needed efforts to scale up evidence-based interventions in LMICs, a framework to routinely assess the quality of care is essential, because providers typically are non-professionals receiving a brief training. This study provides support for using post-training competency assessments, simple tracking of attendance and monitoring participant’s adoption of intervention strategies using a brief mechanisms of action instrument, as a framework for the evaluation, and improvement, of quality of care for the evaluated caregiver support intervention.

Future research is needed to replicate these findings, or establish the role of the quality of care indicators, for other psychological interventions in LMIC—using a sample of service providers that is fully powered for serial mediation analyses. Further, as brief mechanisms of action instruments appear to capture the combination of active ingredients, it opens the opportunity to also use these instruments to see which of the mechanisms is especially potent—a research strategy that is potentially more feasible compared to dismantling studies. For now, this study provides important preliminary evidence for understanding what makes interventions implemented by non-specialists work in LMIC—offering guidance for monitoring and improve impact in future scaling efforts.

## Supplementary Information

Supplementary Material 1.

## Data Availability

The datasets used and/or analysed during the current study are available from the corresponding author and are stored on a secured site hosted by War Child Alliance.
