# Supplementary material for: Predictors of caregiver support intervention outcomes for refugees in Jordan: a three-path mediational study
Source: Child Adolesc Psychiatry Ment Health. 2025 Dec 7;19:137. doi: 10.1186/s13034-025-00995-1 (PMC12687550; doi:10.1186/s13034-025-00995-1)
Supplement: Supplementary file 1 — Supplementary Material 1. [file 13034_2025_995_MOESM1_ESM.docx]

**Additional File 1. Correlations**

Partial correlations (conditioned on baseline scores) between the outcomes and indicators

| outcome | % attendance total | ENACT mean levels 2, 3, 4 | Skills Use total |
| --- | --- | --- | --- |
| Parenting | 0.101^*^ | 0.030 | 0.389^***^ |
| Parental warmth & responsiveness | 0.103^*^ | 0.041 | 0.873^***^ |
| Harsh parenting | -0.041 | -0.003 | -0.756^***^ |
| Distress (K10) | -0.054 | 0.021 | -0.231^***^ |
| KINDL caregiver-report | 0.041 | -0.094^**^ | 0.385^***^ |
| Wellbeing | 0.127^**^ | -0.011 | 0.370^***^ |
| KINDL self-report | -0.018 | -0.079 | 0.307^***^ |

Note: ** p<.05, **p<.01, ***p<.001*

**Additional File 2. Correlations including suppressors**

Partial correlations (conditioned on baseline scores and including suppressor variables) between the outcomes and indicators

| outcome | % attendance total | ENACT mean levels 2, 3, 4 |
| --- | --- | --- |
| Parenting | 0.091^*^ | 0.009 |
| Parental warmth & responsiveness | 0.099^**^ | 0.017 |
| Harsh parenting | -0.036 | 0.003 |
| Distress (K10) | -0.048 | 0.035 |
| KINDL caregiver-report | 0.063 | -0.094^**^ |
| Wellbeing | 0.114^**^ | -0.046 |
| KINDL self-report | -0.002 | -0.066 |

Note: ** p<.05, **p<.01, ***p<.001*

**Additional File 3. Moderator results**

Below we give for each outcome the model summaries of the regression models to examine the changes in explained variance (R^2^ change) of the model including the moderators compared to the null model. In the tables below, H0 is the null model with only attendance and baseline score of the corresponding outcome and H1 is the null model plus all the moderator main effects, and interactions with attendance. Only when the H1 model explains significantly more variance than the H0, we interpret the regression coefficients.

The moderators included are: caregiver age, gender, nationality, educational level, perception of financial situation, and total number of persons in the household. Attendance was included as the percentage attendance over the 9 sessions. and Competencies was included as the mean competencies score over the levels 2, 3, and 4 post-training. This is because level 1 indicates possible harmful behavior and does not measure competencies like the levels 2 (no competence), 3 (basic competence) and 4 (mastery).

| *Model Summary - Parenting_Total_T2* | | | | | | | | | | | | | | | | | | | |
| --- | --- | --- | --- | --- | --- | --- | --- | --- | --- | --- | --- | --- | --- | --- | --- | --- | --- | --- | --- |
| Model | | R | | R² | | Adjusted R² | | RMSE | | R² Change | | F Change | | df1 | | df2 | | p | |
| H₀ |  | 0.610 |  | 0.373 |  | 0.370 |  | 4.123 |  | 0.373 |  | 149.717 |  | 2 |  | 504 |  | < .001 |  |
| H₁ |  | 0.624 |  | 0.390 |  | 0.370 |  | 4.125 |  | **0.017** |  | **0.847** |  | **16** |  | **490** |  | **0.631** |  |
|  | | | | | | | | | | | | | | | | | | | |
| *Note.*  Null model includes Parenting_Total_T1, att_percentage | | | | | | | | | | | | | | | | | | | |

| *Model Summary - Parental_Warmth&Responsiveness_T2* | | | | | | | | | | | | | | | | | | | |
| --- | --- | --- | --- | --- | --- | --- | --- | --- | --- | --- | --- | --- | --- | --- | --- | --- | --- | --- | --- |
| Model | | R | | R² | | Adjusted R² | | RMSE | | R² Change | | F Change | | df1 | | df2 | | p | |
| H₀ |  | 0.562 |  | 0.316 |  | 0.313 |  | 3.149 |  | 0.316 |  | 116.293 |  | 2 |  | 504 |  | < .001 |  |
| H₁ |  | 0.578 |  | 0.334 |  | 0.312 |  | 3.150 |  | **0.018** |  | **0.848** |  | **16** |  | **490** |  | **0.631** |  |
|  | | | | | | | | | | | | | | | | | | | |
| *Note.*  Null model includes Parental_WR_T1, att_percentage | | | | | | | | | | | | | | | | | | | |

| *Model Summary - Harsh_Parenting_T2* | | | | | | | | | | | | | | | | | | | |
| --- | --- | --- | --- | --- | --- | --- | --- | --- | --- | --- | --- | --- | --- | --- | --- | --- | --- | --- | --- |
| Model | | R | | R² | | Adjusted R² | | RMSE | | R² Change | | F Change | | df1 | | df2 | | p | |
| H₀ |  | 0.505 |  | 0.256 |  | 0.253 |  | 2.009 |  | 0.256 |  | 86.494 |  | 2 |  | 504 |  | < .001 |  |
| H₁ |  | 0.523 |  | 0.274 |  | 0.250 |  | 2.013 |  | **0.018** |  | **0.763** |  | **16** |  | **490** |  | **0.728** |  |
|  | | | | | | | | | | | | | | | | | | | |
| *Note.*  Null model includes Harsh_Parenting_T1, att_percentage | | | | | | | | | | | | | | | | | | | |

| *Model Summary - Distress_Total_T2* | | | | | | | | | | | | | | | | | |
| --- | --- | --- | --- | --- | --- | --- | --- | --- | --- | --- | --- | --- | --- | --- | --- | --- | --- |
| Model | | R | | R² | | Adjusted R² | | RMSE | | R² Change | | df1 | | df2 | | p | |
| M₀ |  | 0.533 |  | 0.284 |  | 0.281 |  | 7.726 |  | 0.284 |  | 2 |  | 504 |  | < .001 |  |
| M₁ |  | 0.574 |  | 0.330 |  | 0.308 |  | 7.581 |  | **0.046** |  | **16** |  | **490** |  | **0.008** |  |
|  | | | | | | | | | | | | | | | | | |
| *Note.*  M₀ includes K10_Total_T1, att_percentage | | | | | | | | | | | | | | | | | |

| Coefficients | | | | | | | | | | | | | |
| --- | --- | --- | --- | --- | --- | --- | --- | --- | --- | --- | --- | --- | --- |
| Model | |  | | Unstandardized | | Standard Error | | Standardizedᵃ | | t | | p | |
| M₀ |  | (Intercept) |  | 11.976 |  | 1.287 |  |  |  | 9.308 |  | < .001 |  |
|  |  | K10_Total_T1 |  | 0.517 |  | 0.037 |  | 0.533 |  | 14.137 |  | < .001 |  |
|  |  | att_percentage |  | -0.013 |  | 0.010 |  | -0.046 |  | -1.218 |  | 0.224 |  |
| M₁ |  | (Intercept) |  | 11.166 |  | 10.125 |  |  |  | 1.103 |  | 0.271 |  |
|  |  | K10_Total_T1 |  | 0.486 |  | 0.039 |  | 0.501 |  | 12.327 |  | < .001 |  |
|  |  | att_percentage |  | 0.019 |  | 0.092 |  | 0.071 |  | 0.211 |  | 0.833 |  |
|  |  | cg_age_t1 |  | 0.218 |  | 0.109 |  | 0.199 |  | 1.990 |  | 0.047 |  |
|  |  | cg_edu_level_t1 |  | -0.373 |  | 0.666 |  | -0.052 |  | -0.560 |  | 0.576 |  |
|  |  | cg_perception_finance_t1 |  | 0.488 |  | 1.090 |  | 0.039 |  | 0.448 |  | 0.655 |  |
|  |  | num_household_children_t1 |  | -0.488 |  | 0.544 |  | -0.088 |  | -0.897 |  | 0.370 |  |
|  |  | cg_gender_t1 (2) |  | -7.265 |  | 1.988 |  |  |  | -3.654 |  | < .001 |  |
|  |  | cg_nationality_t1 (2) |  | -2.516 |  | 7.983 |  |  |  | -0.315 |  | 0.753 |  |
|  |  | cg_nationality_t1 (3) |  | -9.262 |  | 9.668 |  |  |  | -0.958 |  | 0.339 |  |
|  |  | cg_nationality_t1 (5) |  | -2.484 |  | 7.717 |  |  |  | -0.322 |  | 0.748 |  |
|  |  | att_percentage  ✻   cg_age_t1 |  | -0.003 |  | 0.001 |  | -0.398 |  | -1.760 |  | 0.079 |  |
|  |  | att_percentage  ✻   cg_edu_level_t1 |  | -0.006 |  | 0.009 |  | -0.079 |  | -0.593 |  | 0.554 |  |
|  |  | att_percentage  ✻   cg_perception_finance_t1 |  | 0.003 |  | 0.015 |  | 0.036 |  | 0.175 |  | 0.861 |  |
|  |  | att_percentage  ✻   num_household_children_t1 |  | 0.013 |  | 0.007 |  | 0.262 |  | 1.818 |  | 0.070 |  |
|  |  | **att_percentage  ✻   cg_gender_t1 (2)** |  | **0.096** |  | **0.030** |  |  |  | **3.181** |  | **0.002** |  |
|  |  | att_percentage  ✻   cg_nationality_t1 (2) |  | -0.011 |  | 0.028 |  |  |  | -0.410 |  | 0.682 |  |
|  |  | att_percentage  ✻   cg_nationality_t1 (3) |  | NaN |  | NaN |  |  |  | NaN |  | NaN |  |
|  |  | att_percentage  ✻   cg_nationality_t1 (5) |  | NaN |  | NaN |  |  |  | NaN |  | NaN |  |
|  | | | | | | | | | | | | | |
| *Note.  Missing coefficients are undefined because of singularities. Check the data for anything out of order!* | | | | | | | | | | | | | |
| *ᵃ Standardized coefficients can only be computed for continuous predictors.* | | | | | | | | | | | | | |

| *Model Summary – Child wellbeing CG report_Total_T2* | | | | | | | | | | | | | | | | | | | |
| --- | --- | --- | --- | --- | --- | --- | --- | --- | --- | --- | --- | --- | --- | --- | --- | --- | --- | --- | --- |
| Model | | R | | R² | | Adjusted R² | | RMSE | | R² Change | | F Change | | df1 | | df2 | | p | |
| H₀ |  | 0.478 |  | 0.229 |  | 0.226 |  | 11.636 |  | 0.229 |  | 74.748 |  | 2 |  | 504 |  | < .001 |  |
| H₁ |  | 0.491 |  | 0.241 |  | 0.217 |  | 11.704 |  | **0.013** |  | **0.509** |  | **16** |  | **490** |  | **0.943** |  |
|  | | | | | | | | | | | | | | | | | | | |
| *Note.*  Null model includes KINDL_Total_T1, att_percentage | | | | | | | | | | | | | | | | | | | |

| *Model Summary – CG wellbeing_Total_T2* | | | | | | | | | | | | | | | | | | | |
| --- | --- | --- | --- | --- | --- | --- | --- | --- | --- | --- | --- | --- | --- | --- | --- | --- | --- | --- | --- |
| Model | | R | | R² | | Adjusted R² | | RMSE | | R² Change | | F Change | | df1 | | df2 | | p | |
| H₀ |  | 0.493 |  | 0.243 |  | 0.240 |  | 8.586 |  | 0.243 |  | 81.049 |  | 2 |  | 504 |  | < .001 |  |
| H₁ |  | 0.515 |  | 0.265 |  | 0.241 |  | 8.581 |  | **0.022** |  | **0.909** |  | **16** |  | **490** |  | **0.558** |  |
|  | | | | | | | | | | | | | | | | | | | |
| *Note.*  Null model includes WEMBWS_Total_T1, att_percentage | | | | | | | | | | | | | | | | | | | |

Below we give for each outcome the model summaries of the regression models to examine the changes in explained variance (R^2^ change) of the model including the moderators compared to the null model. In the tables below, H0 is the null model with only competencies and baseline score of the corresponding outcome and H1 is the null model plus all the moderator main effects, and interactions with competencies. Only when the H1 model explains significantly more variance than the H0, we interpret the regression coefficients.

The moderators included are: caregiver age, gender, nationality, educational level, perception of financial situation, and total number of persons in the household. Competencies was included as the mean competencies score over the levels 2, 3, and 4 post-training. This is because level 1 indicates possible harmful behavior and does not measure competencies in the same scale as the levels 2 (no competence), 3 (basic competence) and 4 (mastery). Therefore, the same models were additionally run with competencies included as only the harmful attributes and only the helpful attributes.

| *Model Summary - Parenting_Total_T2* | | | | | | | | | | | | | | | | | | | |
| --- | --- | --- | --- | --- | --- | --- | --- | --- | --- | --- | --- | --- | --- | --- | --- | --- | --- | --- | --- |
| Model | | R | | R² | | Adjusted R² | | RMSE | | R² Change | | F Change | | df1 | | df2 | | p | |
| H₀ |  | 0.612 |  | 0.374 |  | 0.372 |  | 4.147 |  | 0.374 |  | 146.764 |  | 2 |  | 491 |  | < .001 |  |
| H₁ |  | 0.627 |  | 0.393 |  | 0.371 |  | 4.148 |  | **0.019** |  | **0.908** |  | **16** |  | **476** |  | **0.560** |  |
|  | | | | | | | | | | | | | | | | | | | |
| *Note.*  Null model includes Parenting_Total_T1, ENACTpost_total_234mean | | | | | | | | | | | | | | | | | | | |

| *Model Summary - Parental_Warmth&Responsiveness_T2* | | | | | | | | | | | | | | | | | | | |
| --- | --- | --- | --- | --- | --- | --- | --- | --- | --- | --- | --- | --- | --- | --- | --- | --- | --- | --- | --- |
| Model | | R | | R² | | Adjusted R² | | RMSE | | R² Change | | F Change | | df1 | | df2 | | p | |
| H₀ |  | 0.570 |  | 0.325 |  | 0.322 |  | 3.147 |  | 0.325 |  | 118.285 |  | 2 |  | 491 |  | < .001 |  |
| H₁ |  | 0.589 |  | 0.347 |  | 0.324 |  | 3.143 |  | **0.022** |  | **1.008** |  | **16** |  | **476** |  | **0.446** |  |
|  | | | | | | | | | | | | | | | | | | | |
| *Note.*  Null model includes Parental_WR_T1, ENACTpost_total_234mean | | | | | | | | | | | | | | | | | | | |

| *Model Summary – Harsh_Parenting_T2* | | | | | | | | | | | | | | | | | | | |
| --- | --- | --- | --- | --- | --- | --- | --- | --- | --- | --- | --- | --- | --- | --- | --- | --- | --- | --- | --- |
| Model | | R | | R² | | Adjusted R² | | RMSE | | R² Change | | F Change | | df1 | | df2 | | p | |
| H₀ |  | 0.506 |  | 0.257 |  | 0.254 |  | 2.008 |  | 0.257 |  | 84.713 |  | 2 |  | 491 |  | < .001 |  |
| H₁ |  | 0.532 |  | 0.283 |  | 0.257 |  | 2.004 |  | **0.026** |  | **1.082** |  | **16** |  | **476** |  | **0.369** |  |
|  | | | | | | | | | | | | | | | | | | | |
| *Note.*  Null model includes Harsh_Parenting_T1, ENACTpost_total_234mean | | | | | | | | | | | | | | | | | | | |

| *Model Summary – Distress_Total_T2* | | | | | | | | | | | | | | | | | | | |
| --- | --- | --- | --- | --- | --- | --- | --- | --- | --- | --- | --- | --- | --- | --- | --- | --- | --- | --- | --- |
| Model | | R | | R² | | Adjusted R² | | RMSE | | R² Change | | F Change | | df1 | | df2 | | p | |
| H₀ |  | 0.535 |  | 0.287 |  | 0.284 |  | 7.739 |  | 0.287 |  | 98.623 |  | 2 |  | 491 |  | < .001 |  |
| H₁ |  | 0.565 |  | 0.319 |  | 0.295 |  | 7.677 |  | **0.033** |  | **1.432** |  | **16** |  | **476** |  | **0.122** |  |
|  | | | | | | | | | | | | | | | | | | | |
| *Note.*  Null model includes K10_Total_T1, ENACTpost_total_234mean | | | | | | | | | | | | | | | | | | | |

| *Model Summary – Child wellbeing CG report_Total_T2* | | | | | | | | | | | | | | | | | | | |
| --- | --- | --- | --- | --- | --- | --- | --- | --- | --- | --- | --- | --- | --- | --- | --- | --- | --- | --- | --- |
| Model | | R | | R² | | Adjusted R² | | RMSE | | R² Change | | F Change | | df1 | | df2 | | p | |
| H₀ |  | 0.495 |  | 0.245 |  | 0.242 |  | 11.561 |  | 0.245 |  | 79.738 |  | 2 |  | 491 |  | < .001 |  |
| H₁ |  | 0.504 |  | 0.254 |  | 0.228 |  | 11.671 |  | **0.009** |  | **0.362** |  | **16** |  | **476** |  | **0.990** |  |
|  | | | | | | | | | | | | | | | | | | | |
| *Note.*  Null model includes KINDL_Total_T1, ENACTpost_total_234mean | | | | | | | | | | | | | | | | | | | |

| *Model Summary – CG wellbeing_Total_T2* | | | | | | | | | | | | | | | | | | | |
| --- | --- | --- | --- | --- | --- | --- | --- | --- | --- | --- | --- | --- | --- | --- | --- | --- | --- | --- | --- |
| Model | | R | | R² | | Adjusted R² | | RMSE | | R² Change | | F Change | | df1 | | df2 | | p | |
| H₀ |  | 0.475 |  | 0.226 |  | 0.222 |  | 8.659 |  | 0.226 |  | 71.518 |  | 2 |  | 491 |  | < .001 |  |
| H₁ |  | 0.505 |  | 0.255 |  | 0.229 |  | 8.623 |  | **0.030** |  | **1.194** |  | **16** |  | **476** |  | **0.268** |  |
|  | | | | | | | | | | | | | | | | | | | |

*Note.*  Null model includes WEMBWS_Total_T1, ENACTpost_total_234mean

The tables below give the same models as above, but with competencies defined as the number of harmful attributes.

| *Model Summary - Parenting_Total_T2* | | | | | | | | | | | | | | | | | |
| --- | --- | --- | --- | --- | --- | --- | --- | --- | --- | --- | --- | --- | --- | --- | --- | --- | --- |
| Model | | R | | R² | | Adjusted R² | | RMSE | | R² Change | | df1 | | df2 | | p | |
| M₀ |  | 0.613 |  | 0.376 |  | 0.373 |  | 4.140 |  | 0.376 |  | 2 |  | 491 |  | < .001 |  |
| M₁ |  | 0.630 |  | 0.397 |  | 0.375 |  | 4.135 |  | **0.021** |  | **16** |  | **476** |  | **0.436** |  |
|  | | | | | | | | | | | | | | | | | |
| *Note.*  M₀ includes Parenting_Total_T1, ENACTpost_harm_att | | | | | | | | | | | | | | | | | |

| *Model Summary - Parental_Warmth&Responsiveness_T2* | | | | | | | | | | | | | | | | | |
| --- | --- | --- | --- | --- | --- | --- | --- | --- | --- | --- | --- | --- | --- | --- | --- | --- | --- |
| Model | | R | | R² | | Adjusted R² | | RMSE | | R² Change | | df1 | | df2 | | p | |
| M₀ |  | 0.571 |  | 0.326 |  | 0.323 |  | 3.145 |  | 0.326 |  | 2 |  | 491 |  | < .001 |  |
| M₁ |  | 0.590 |  | 0.348 |  | 0.324 |  | 3.142 |  | **0.022** |  | **16** |  | **476** |  | **0.464** |  |
|  | | | | | | | | | | | | | | | | | |
| *Note.*  M₀ includes Parental_WR_T1, ENACTpost_harm_att | | | | | | | | | | | | | | | | | |

| *Model Summary - Harsh_Parenting_T2* | | | | | | | | | | | | | | | | | |
| --- | --- | --- | --- | --- | --- | --- | --- | --- | --- | --- | --- | --- | --- | --- | --- | --- | --- |
| Model | | R | | R² | | Adjusted R² | | RMSE | | R² Change | | df1 | | df2 | | p | |
| M₀ |  | 0.508 |  | 0.258 |  | 0.255 |  | 2.006 |  | 0.258 |  | 2 |  | 491 |  | < .001 |  |
| M₁ |  | 0.531 |  | 0.282 |  | 0.256 |  | 2.004 |  | **0.024** |  | **16** |  | **476** |  | **0.466** |  |
|  | | | | | | | | | | | | | | | | | |
| *Note.*  M₀ includes Harsh_Parenting_T1, ENACTpost_harm_att | | | | | | | | | | | | | | | | | |

| *Model Summary - Distress_Total_T2* | | | | | | | | | | | | | | | | | |
| --- | --- | --- | --- | --- | --- | --- | --- | --- | --- | --- | --- | --- | --- | --- | --- | --- | --- |
| Model | | R | | R² | | Adjusted R² | | RMSE | | R² Change | | df1 | | df2 | | p | |
| M₀ |  | 0.537 |  | 0.288 |  | 0.286 |  | 7.729 |  | 0.288 |  | 2 |  | 491 |  | < .001 |  |
| M₁ |  | 0.558 |  | 0.312 |  | 0.287 |  | 7.720 |  | **0.023** |  | **16** |  | **476** |  | **0.446** |  |
|  | | | | | | | | | | | | | | | | | |
| *Note.*  M₀ includes K10_Total_T1, ENACTpost_harm_att | | | | | | | | | | | | | | | | | |

| *Model Summary - Child wellbeing CG report_Total_T2* | | | | | | | | | | | | | | | | | |
| --- | --- | --- | --- | --- | --- | --- | --- | --- | --- | --- | --- | --- | --- | --- | --- | --- | --- |
| Model | | R | | R² | | Adjusted R² | | RMSE | | R² Change | | df1 | | df2 | | p | |
| M₀ |  | 0.493 |  | 0.243 |  | 0.240 |  | 11.577 |  | 0.243 |  | 2 |  | 491 |  | < .001 |  |
| M₁ |  | 0.512 |  | 0.262 |  | 0.236 |  | 11.608 |  | **0.019** |  | **16** |  | **476** |  | **0.715** |  |
|  | | | | | | | | | | | | | | | | | |
| *Note.*  M₀ includes KINDL_Total_T1, ENACTpost_harm_att | | | | | | | | | | | | | | | | | |

| *Model Summary - CG wellbeing_Total_T2* | | | | | | | | | | | | | | | | | |
| --- | --- | --- | --- | --- | --- | --- | --- | --- | --- | --- | --- | --- | --- | --- | --- | --- | --- |
| Model | | R | | R² | | Adjusted R² | | RMSE | | R² Change | | df1 | | df2 | | p | |
| M₀ |  | 0.480 |  | 0.230 |  | 0.227 |  | 8.633 |  | 0.230 |  | 2 |  | 491 |  | < .001 |  |
| M₁ |  | 0.504 |  | 0.254 |  | 0.227 |  | 8.631 |  | **0.024** |  | **16** |  | **476** |  | **0.510** |  |
|  | | | | | | | | | | | | | | | | | |
| *Note.*  M₀ includes WEMBWS_Total_T1, ENACTpost_harm_att | | | | | | | | | | | | | | | | | |

The tables below give the same models as above, but with competencies defined as the number of helpful attributes.

| *Model Summary - Parenting_Total_T2* | | | | | | | | | | | | | | | | | |
| --- | --- | --- | --- | --- | --- | --- | --- | --- | --- | --- | --- | --- | --- | --- | --- | --- | --- |
| Model | | R | | R² | | Adjusted R² | | RMSE | | R² Change | | df1 | | df2 | | p | |
| M₀ |  | 0.612 |  | 0.375 |  | 0.372 |  | 4.145 |  | 0.375 |  | 2 |  | 491 |  | < .001 |  |
| M₁ |  | 0.628 |  | 0.394 |  | 0.372 |  | 4.144 |  | **0.019** |  | **16** |  | **476** |  | **0.508** |  |
|  | | | | | | | | | | | | | | | | | |
| *Note.*  M₀ includes Parenting_Total_T1, ENACTpost_help_att | | | | | | | | | | | | | | | | | |

| *Model Summary - Parental_Warmth&Responsiveness_T2* | | | | | | | | | | | | | | | | | |
| --- | --- | --- | --- | --- | --- | --- | --- | --- | --- | --- | --- | --- | --- | --- | --- | --- | --- |
| Model | | R | | R² | | Adjusted R² | | RMSE | | R² Change | | df1 | | df2 | | p | |
| M₀ |  | 0.570 |  | 0.325 |  | 0.323 |  | 3.146 |  | 0.325 |  | 2 |  | 491 |  | < .001 |  |
| M₁ |  | 0.590 |  | 0.348 |  | 0.325 |  | 3.142 |  | **0.022** |  | **16** |  | **476** |  | **0.430** |  |
|  | | | | | | | | | | | | | | | | | |
| *Note.*  M₀ includes Parental_WR_T1, ENACTpost_help_att | | | | | | | | | | | | | | | | | |

| *Model Summary - Harsh_Parenting_T2* | | | | | | | | | | | | | | | | | |
| --- | --- | --- | --- | --- | --- | --- | --- | --- | --- | --- | --- | --- | --- | --- | --- | --- | --- |
| Model | | R | | R² | | Adjusted R² | | RMSE | | R² Change | | df1 | | df2 | | p | |
| M₀ |  | 0.507 |  | 0.257 |  | 0.254 |  | 2.008 |  | 0.257 |  | 2 |  | 491 |  | < .001 |  |
| M₁ |  | 0.529 |  | 0.280 |  | 0.254 |  | 2.007 |  | **0.023** |  | **16** |  | **476** |  | **0.509** |  |
|  | | | | | | | | | | | | | | | | | |
| *Note.*  M₀ includes Harsh_Parenting_T1, ENACTpost_help_att | | | | | | | | | | | | | | | | | |

| *Model Summary - Distress_Total_T2* | | | | | | | | | | | | | | | | | |
| --- | --- | --- | --- | --- | --- | --- | --- | --- | --- | --- | --- | --- | --- | --- | --- | --- | --- |
| Model | | R | | R² | | Adjusted R² | | RMSE | | R² Change | | df1 | | df2 | | p | |
| M₀ |  | 0.535 |  | 0.286 |  | 0.284 |  | 7.739 |  | 0.286 |  | 2 |  | 491 |  | < .001 |  |
| M₁ |  | 0.568 |  | 0.323 |  | 0.299 |  | 7.656 |  | **0.037** |  | **16** |  | **476** |  | **0.063** |  |
|  | | | | | | | | | | | | | | | | | |
| *Note.*  M₀ includes K10_Total_T1, ENACTpost_help_att | | | | | | | | | | | | | | | | | |

| *Model Summary - Child wellbeing CG report_Total_T2* | | | | | | | | | | | | | | | | | |
| --- | --- | --- | --- | --- | --- | --- | --- | --- | --- | --- | --- | --- | --- | --- | --- | --- | --- |
| Model | | R | | R² | | Adjusted R² | | RMSE | | R² Change | | df1 | | df2 | | p | |
| M₀ |  | 0.493 |  | 0.243 |  | 0.240 |  | 11.580 |  | 0.243 |  | 2 |  | 491 |  | < .001 |  |
| M₁ |  | 0.506 |  | 0.256 |  | 0.230 |  | 11.654 |  | **0.014** |  | **16** |  | **476** |  | **0.923** |  |
|  | | | | | | | | | | | | | | | | | |
| *Note.*  M₀ includes KINDL_Total_T1, ENACTpost_help_att | | | | | | | | | | | | | | | | | |

| *Model Summary - CG wellbeing_Total_T2* | | | | | | | | | | | | | | | | | |
| --- | --- | --- | --- | --- | --- | --- | --- | --- | --- | --- | --- | --- | --- | --- | --- | --- | --- |
| Model | | R | | R² | | Adjusted R² | | RMSE | | R² Change | | df1 | | df2 | | p | |
| M₀ |  | 0.475 |  | 0.226 |  | 0.222 |  | 8.659 |  | 0.226 |  | 2 |  | 491 |  | < .001 |  |
| M₁ |  | 0.510 |  | 0.260 |  | 0.233 |  | 8.599 |  | **0.034** |  | **16** |  | **476** |  | **0.153** |  |
|  | | | | | | | | | | | | | | | | | |
| *Note.*  M₀ includes WEMBWS_Total_T1, ENACTpost_help_att | | | | | | | | | | | | | | | | | |

**Additional File 4. CSI Fidelity Tool**

The following are 9 tables corresponding to 9 fidelity checklists for each session of the Caregiver Support Intervention (CSI). Each checklist is to be filled by both facilitator and co-facilitator of a CSI group to assess their fidelity in delivering the sessions from the manual.

| **Session Number:** | | 1 | **Date:** |  | | | |  |  |  |
| --- | --- | --- | --- | --- | --- | --- | --- | --- | --- | --- |
| **Number of Participants:** | |  | **Sex:** |  | | **Nationality:** | |  | |  |
| **Number of Absentees:** | |  | **Reasons:** |  | | | | | |  |
| **Are there any dropout?** | |  | **Reasons:** |  | | | | | |  |
| **Session title:** | | Introduction and Group Building | | | | | | | |  |
| **Facilitator Name:** | |  | | **Co-facilitator name:** | | | |  | |  |
|  |  |  |  |  |  |  |  |  |  |  |
|  |  |  |  |  |  |  |  |  |  |  |
|  | | | | **Yes** | **No** | **N/A** | **Comments** | | | |
| **1** | **Were all the resources ready for the session?** | | |  |  |  |  | | | |
| **2** | **Did you welcome the participants?** | | |  |  |  |  | | | |
| **3** | *Did you say the welcoming sentence as it is or in a very similar way?* | | |  |  |  |  | | | |
| **4** | **Did you do Exercise 1 "Name Game with Ball"?** | | |  |  |  |  | | | |
| **5** | **Did you talk about the logistics/ practicalities?** | | |  |  |  |  | | | |
| **6** | *Did you say the logistics/practicalities sentence as it is or in a very similar way?* | | |  |  |  |  | | | |
| **7** | **Did you suggest to make rules and guidelines together as a group?** | | |  |  |  |  | | | |
| **8** | *Did you add our own rules and guidelines?* | | |  |  |  |  | | | |
| **9** | *Did you say the connection point as it is or in a very similar way?* | | |  |  |  |  | | | |
| **10** | **Did you do Exercise 2 "Expectations, goals and concerns"?** | | |  |  |  |  | | | |
| **11** | **Did you introduce goals & structure, Home Practice?** | | |  |  |  |  | | | |
| **12** | **Did you do Exercise 3 "Getting to know each other better"?** | | |  |  |  |  | | | |
| **13** | *Did you say the connection point as it is or in a very similar way?* | | |  |  |  |  | | | |
| **14** | **Did you do Exercise 4 "Stories"?** | | |  |  |  |  | | | |
| **15** | *Did you say the connection point as it is or in a very similar way?* | | |  |  |  |  | | | |
| **16** | **Did you do the relaxation and stress reduction exercise "Counting the Breath"?** | | |  |  |  |  | | | |
| **17** | **Did you introduce home practice?** | | |  |  |  |  | | | |
| **18** | **Did you assign the home practice?** | | |  |  |  |  | | | |
| **19** | **Did you check-out?** | | |  |  |  |  | | | |

| **Session Number:** | 2 | **Date:** |  | | | |  |
| --- | --- | --- | --- | --- | --- | --- | --- |
| **Number of Participants:** |  | **Sex:** |  | | **Nationality:** |  |  |
| **Number of Absentees:** |  | **Reasons:** |  | | | |  |
| **Are there any dropout?** |  | **Reasons:** |  | | | |  |
| **Session title:** | Our Sources of Strength | | | | | |  |
| **Facilitator Name:** |  | | **Co-facilitator name:** | | |  |  |
|  | | | | | | | |
|  | | | **Yes** | **No** | **N/A** | **Comments** | |
| **1** | **Were all the resources ready for the session?** | |  |  |  |  | |
| **2** | **Did you welcome the participants?** | |  |  |  |  | |
| **3** | *Did you say the welcoming sentence as it is or in a very similar way?* | |  |  |  |  | |
| **4** | **Did you do Exercise 1"Name Game with Ball"?** | |  |  |  |  | |
| **5** | **Did you check if they did the Home Practice?** | |  |  |  |  | |
| **6** | **Did you do the group talk "Information on stress"?** | |  |  |  |  | |
| **7** | *Did you follow the exercise from manual as it is?* | |  |  |  |  | |
| **8** | *Did you say the connection point as it is or in a very similar way?* | |  |  |  |  | |
| **9** | **Did you do Exercise 2 "Resource Mapping"?** | |  |  |  |  | |
| **10** | *Did you follow the exercise from manual as it is?* | |  |  |  |  | |
| **11** | *Did you say the connection point as it is or in a very similar way?* | |  |  |  |  | |
| **12** | **Did you do the first relaxation and stress reduction exercise "Body Scan"?** | |  |  |  |  | |
| **13** | *Did you follow the exercise from manual as it is?* | |  |  |  |  | |
| **14** | **Did you assign the home practice?** | |  |  |  |  | |
| **15** | **Did you check-out?** | |  |  |  |  | |
|  | | | | | | | |
| **16** | **Were there any comments or concerns from the caregivers?** | | | | | | |
|  | | | | | | | |
|  |  |  |  |  |  |  |  |
|  |  |  |  |  |  |  |  |
|  |  |  |  |  |  |  |  |
| **17** | **Were there any comments or concerns from the facilitators?** | | | | | | |
|  | | | | | | | |
|  |  |  |  |  |  |  |  |
|  |  |  |  |  |  |  |  |
|  |  |  |  |  |  |  |  |

| **Session Number:** | 3 | **Date:** |  | | | |  |
| --- | --- | --- | --- | --- | --- | --- | --- |
| **Number of Participants:** |  | **Sex:** |  | | **Nationality:** |  |  |
| **Number of Absentees:** |  | **Reasons:** |  | | | |  |
| **Are there any dropout?** |  | **Reasons:** |  | | | |  |
| **Session title:** | Lowering Our Stress | | | | | |  |
| **Facilitator Name:** |  | | **Co-facilitator name:** | | |  |  |
|  | | | | | | | |
|  | | | **Yes** | **No** | **N/A** | **Comments** | |
| **1** | **Were all the resources ready for the session?** | |  |  |  |  | |
| **2** | **Did you welcome the participants?** | |  |  |  |  | |
| **3** | *Did you say the welcoming sentence as it is or in a very similar way?* | |  |  |  |  | |
| **4** | **Did you do Exercise 1 "Red ball, green ball"?** | |  |  |  |  | |
| **5** | **Did you check if they did the Home Practice?** | |  |  |  |  | |
| **6** | *Did you follow the exercise from manual as it is?* | |  |  |  |  | |
| **7** | *Did you say the connection point as it is or in a very similar way?* | |  |  |  |  | |
| **8** | **Did you do Exercise 2 "Thinking too Much"?** | |  |  |  |  | |
| **9** | *Did you follow the exercise from manual as it is?* | |  |  |  |  | |
| **10** | *Did you say the connection point as it is or in a very similar way?* | |  |  |  |  | |
| **11** | **Did you do the first relaxation and stress reduction exercise "Stepping Back From our Thoughts"?** | |  |  |  |  | |
| **12** | *Did you follow the exercise from manual as it is?* | |  |  |  |  | |
| **13** | **Did you do the second relaxation and stress reduction exercise "Grounding"?** | |  |  |  |  | |
| **14** | *Did you follow the exercise from manual as it is?* | |  |  |  |  | |
| **15** | **Did you assign the home practice?** | |  |  |  |  | |
| **16** | **Did you check-out?** | |  |  |  |  | |
|  | | | | | | | |
| **17** | **Were there any comments or concerns from the caregivers?** | | | | | | |
|  | | | | | | | |
|  |  |  |  |  |  |  |  |
|  |  |  |  |  |  |  |  |
|  |  |  |  |  |  |  |  |
| **18** | **Were there any comments or concerns from the facilitators?** | | | | | | |
|  | | | | | | | |
|  |  |  |  |  |  |  |  |
|  |  |  |  |  |  |  |  |
|  |  |  |  |  |  |  |  |

| **Session Number:** | 4 | **Date:** |  | | | |  |
| --- | --- | --- | --- | --- | --- | --- | --- |
| **Number of Participants:** |  | **Sex:** |  | | **Nationality:** |  |  |
| **Number of Absentees:** |  | **Reasons:** |  | | | |  |
| **Are there any dropout?** |  | **Reasons:** |  | | | |  |
| **Session title:** | Coping with Frustration and Anger | | | | | |  |
| **Facilitator Name:** |  | | **Co-facilitator name:** | | |  |  |
|  | | | | | | | |
|  | | | **Yes** | **No** | **N/A** | **Comments** | |
| **1** | **Were all the resources ready for the session?** | |  |  |  |  | |
| **2** | **Did you welcome the participants?** | |  |  |  |  | |
| **3** | *Did you say the welcoming sentence as it is or in a very similar way?* | |  |  |  |  | |
| **4** | **Did you do Exercise 1 "The Accepting Circle"?** | |  |  |  |  | |
| **5** | **Did you check if they did the Home Practice?** | |  |  |  |  | |
| **6** | **Did you do the group talk "Dealing with anger and frustration"?** | |  |  |  |  | |
| **7** | *Did you follow the exercise from manual as it is?* | |  |  |  |  | |
| **8** | *Did you say the connection point as it is or in a very similar way?* | |  |  |  |  | |
| **9** | **Did you do Exercise 2 "Anger: Triggers and Coping Strategies"?** | |  |  |  |  | |
| **10** | *Did you follow the exercise from manual as it is?* | |  |  |  |  | |
| **11** | *Did you say the connection point as it is or in a very similar way?* | |  |  |  |  | |
| **12** | **Did you do Exercise 3 "Role Plays"?** | |  |  |  |  | |
| **13** | *Did you follow the exercise from manual as it is?* | |  |  |  |  | |
| **14** | *Did you say the connection point as it is or in a very similar way?* | |  |  |  |  | |
| **15** | **Did you do the first relaxation and stress reduction exercise "Relaxing through the senses"?** | |  |  |  |  | |
| **16** | *Did you follow the exercise from manual as it is?* | |  |  |  |  | |
| **17** | **Did you assign the home practice?** | |  |  |  |  | |
| **18** | **Did you check-out?** | |  |  |  |  | |
|  | | | | | | | |
| **19** | **Were there any comments or concerns from the caregivers?** | | | | | | |
|  | | | | | | | |
|  |  |  |  |  |  |  |  |
|  |  |  |  |  |  |  |  |
|  |  |  |  |  |  |  |  |
| **20** | **Were there any comments or concerns from the facilitators?** | | | | | | |
|  | | | | | | | |
|  |  |  |  |  |  |  |  |
|  |  |  |  |  |  |  |  |
|  |  |  |  |  |  |  |  |

| **Session Number:** | 5 | **Date:** |  | | | |  |
| --- | --- | --- | --- | --- | --- | --- | --- |
| **Number of Participants:** |  | **Sex:** |  | | **Nationality:** |  |  |
| **Number of Absentees:** |  | **Reasons:** |  | | | |  |
| **Are there any dropout?** |  | **Reasons:** |  | | | |  |
| **Session title:** | Parental Stress and Parental Influence | | | | | |  |
| **Facilitator Name:** |  | | **Co-facilitator name:** | | |  |  |
|  | | | | | | | |
|  | | | **Yes** | **No** | **N/A** | **Comments** | |
| **1** | **Were all the resources ready for the session?** | |  |  |  |  | |
| **2** | **Did you welcome the participants?** | |  |  |  |  | |
| **3** | *Did you say the welcoming sentence as it is or in a very similar way?* | |  |  |  |  | |
| **4** | **Did you do Exercise 1"A song from the past and present"?** | |  |  |  |  | |
| **5** | **Did you check if they did the Home Practice?** | |  |  |  |  | |
| **6** | *Did you say the connection point as it is or in a very similar way?* | |  |  |  |  | |
| **7** | **Did you do Exercise 2 "Challenges of being a parent during difficult times"?** | |  |  |  |  | |
| **8** | *Did you follow the exercise from manual as it is?* | |  |  |  |  | |
| **9** | *Did you say the connection point as it is or in a very similar way?* | |  |  |  |  | |
| **10** | **Did you do Exercise 3 "Recognizing Our Influence as Parents"?** | |  |  |  |  | |
| **11** | *Did you follow the exercise from manual as it is?* | |  |  |  |  | |
| **12** | *Did you say the connection point as it is or in a very similar way?* | |  |  |  |  | |
| **13** | **Did you do the first relaxation and stress reduction exercise "Stepping Back From Our Thoughts (2nd practice of this technique)"?** | |  |  |  |  | |
| **14** | *Did you follow the exercise from manual as it is?* | |  |  |  |  | |
| **15** | **Did you assign the home practice?** | |  |  |  |  | |
| **16** | **Did you check-out?** | |  |  |  |  | |
|  | | | | | | | |
| **17** | **Were there any comments or concerns from the caregivers?** | | | | | | |
|  | | | | | | | |
|  |  |  |  |  |  |  |  |
|  |  |  |  |  |  |  |  |
|  |  |  |  |  |  |  |  |
| **18** | **Were there any comments or concerns from the facilitators?** | | | | | | |
|  | | | | | | | |
|  |  |  |  |  |  |  |  |
|  |  |  |  |  |  |  |  |
|  |  |  |  |  |  |  |  |

| **Session Number:** | 6 | **Date:** |  | | | |  |
| --- | --- | --- | --- | --- | --- | --- | --- |
| **Number of Participants:** |  | **Sex:** |  | | **Nationality:** |  |  |
| **Number of Absentees:** |  | **Reasons:** |  | | | |  |
| **Are there any dropout?** |  | **Reasons:** |  | | | |  |
| **Session title:** | Increasing Our Influence as Parents - Part I: Positive Attention | | | | | |  |
| **Facilitator Name:** |  | | **Co-facilitator name:** | | |  |  |
|  | | | | | | | |
|  | | | **Yes** | **No** | **N/A** | **Comments** | |
| **1** | **Were all the resources ready for the session?** | |  |  |  |  | |
| **2** | **Did you welcome the participants?** | |  |  |  |  | |
| **3** | *Did you say the welcoming sentence as it is or in a very similar way?* | |  |  |  |  | |
| **4** | **Did you do Exercise 1 "The Mirror Game"?** | |  |  |  |  | |
| **5** | **Did you check if they did the Home Practice?** | |  |  |  |  | |
| **6** | **Did you do Exercise 2 "Strategies for Increasing Parental Influence"?** | |  |  |  |  | |
| **7** | *Did you follow the exercise from manual as it is?* | |  |  |  |  | |
| **8** | *Did you say the connection point as it is or in a very similar way?* | |  |  |  |  | |
| **9** | **Did you do the first relaxation and stress reduction exercise "A Safe Place"?** | |  |  |  |  | |
| **10** | *Did you follow the exercise from manual as it is?* | |  |  |  |  | |
| **11** | **Did you assign the home practice?** | |  |  |  |  | |
| **12** | **Did you check-out?** | |  |  |  |  | |
|  | | | | | | | |
| **13** | **Were there any comments or concerns from the caregivers?** | | | | | | |
|  | | | | | | | |
|  |  |  |  |  |  |  |  |
|  |  |  |  |  |  |  |  |
|  |  |  |  |  |  |  |  |
| **14** | **Were there any comments or concerns from the facilitators?** | | | | | | |
|  | | | | | | | |
|  |  |  |  |  |  |  |  |
|  |  |  |  |  |  |  |  |
|  |  |  |  |  |  |  |  |

| **Session Number:** | 7 | **Date:** |  | | | |  |
| --- | --- | --- | --- | --- | --- | --- | --- |
| **Number of Participants:** |  | **Sex:** |  | | **Nationality:** |  |  |
| **Number of Absentees:** |  | **Reasons:** |  | | | |  |
| **Are there any dropout?** |  | **Reasons:** |  | | | |  |
| **Session title:** | Increasing Our Influence as Parents - Part 2: Effective Discipline | | | | | |  |
| **Facilitator Name:** |  | | **Co-facilitator name:** | | |  |  |
|  | | | | | | | |
|  | | | **Yes** | **No** | **N/A** | **Comments** | |
| **1** | **Were all the resources ready for the session?** | |  |  |  |  | |
| **2** | **Did you welcome the participants?** | |  |  |  |  | |
| **3** | *Did you say the welcoming sentence as it is or in a very similar way?* | |  |  |  |  | |
| **4** | **Did you do Exercise 1 "Basket of Fruits"?** | |  |  |  |  | |
| **5** | **Did you check if they did the Home Practice?** | |  |  |  |  | |
| **6** | **Did you do the group talk "Children and Stress"?** | |  |  |  |  | |
| **7** | *Did you follow the exercise from manual as it is?* | |  |  |  |  | |
| **8** | *Did you say the connection point as it is or in a very similar way?* | |  |  |  |  | |
| **9** | **Did you do Exercise 2?** | |  |  |  |  | |
| **10** | *Did you follow the exercise from manual as it is?* | |  |  |  |  | |
| **11** | *Did you say the connection point as it is or in a very similar way?* | |  |  |  |  | |
| **12** | **Did you do Exercise 3 "Effective Discipline"?** | |  |  |  |  | |
| **13** | *Did you follow the exercise from manual as it is?* | |  |  |  |  | |
| **14** | *Did you say the connection point as it is or in a very similar way?* | |  |  |  |  | |
| **15** | **Did you do the first relaxation and stress reduction exercise "Informal breathing practice"?** | |  |  |  |  | |
| **16** | *Did you follow the exercise from manual as it is?* | |  |  |  |  | |
| **17** | **Did you assign the home practice?** | |  |  |  |  | |
| **18** | **Did you check-out?** | |  |  |  |  | |
|  | | | | | | | |
| **19** | **Were there any comments or concerns from the caregivers?** | | | | | | |
|  | | | | | | | |
|  |  |  |  |  |  |  |  |
|  |  |  |  |  |  |  |  |
|  |  |  |  |  |  |  |  |
| **20** | **Were there any comments or concerns from the facilitators?** | | | | | | |
|  | | | | | | | |
|  |  |  |  |  |  |  |  |
|  |  |  |  |  |  |  |  |
|  |  |  |  |  |  |  |  |

| **Session Number:** | 8 | **Date:** |  | | | |  |
| --- | --- | --- | --- | --- | --- | --- | --- |
| **Number of Participants:** |  | **Sex:** |  | | **Nationality:** |  |  |
| **Number of Absentees:** |  | **Reasons:** |  | | | |  |
| **Are there any dropout?** |  | **Reasons:** |  | | | |  |
| **Session title:** | Positive Parenting: Practice | | | | | |  |
| **Facilitator Name:** |  | | **Co-facilitator name:** | | |  |  |
|  | | | | | | | |
|  | | | **Yes** | **No** | **N/A** | **Comments** | |
| **1** | **Were all the resources ready for the session?** | |  |  |  |  | |
| **2** | **Did you welcome the participants?** | |  |  |  |  | |
| **3** | *Did you say the welcoming sentence as it is or in a very similar way?* | |  |  |  |  | |
| **4** | **Did you do Exercise 1 "Pass Yes"?** | |  |  |  |  | |
| **5** | **Did you check if they did the Home Practice?** | |  |  |  |  | |
| **6** | **Did you do Exercise 2 "Praise"?** | |  |  |  |  | |
| **7** | *Did you follow the exercise from manual as it is?* | |  |  |  |  | |
| **8** | *Did you say the connection point as it is or in a very similar way?* | |  |  |  |  | |
| **9** | **Did you do Exercise 3 "Positive Parenting Practice"?** | |  |  |  |  | |
| **10** | *Did you follow the exercise from manual as it is?* | |  |  |  |  | |
| **11** | *Did you say the connection point as it is or in a very similar way?* | |  |  |  |  | |
| **12** | **Did you do Exercise 4 "The Power of Stories & Storytelling"?** | |  |  |  |  | |
| **13** | *Did you follow the exercise from manual as it is?* | |  |  |  |  | |
| **14** | *Did you say the connection point as it is or in a very similar way?* | |  |  |  |  | |
| **15** | **Did you assign the home practice?** | |  |  |  |  | |
| **16** | **Did you check-out?** | |  |  |  |  | |
|  | | | | | | | |
| **17** | **Were there any comments or concerns from the caregivers?** | | | | | | |
|  | | | | | | | |
|  |  |  |  |  |  |  |  |
|  |  |  |  |  |  |  |  |
|  |  |  |  |  |  |  |  |
| **18** | **Were there any comments or concerns from the facilitators?** | | | | | | |
|  | | | | | | | |
|  |  |  |  |  |  |  |  |
|  |  |  |  |  |  |  |  |
|  |  |  |  |  |  |  |  |

| **Session Number:** | 9 | **Date:** |  | | | |  |
| --- | --- | --- | --- | --- | --- | --- | --- |
| **Number of Participants:** |  | **Sex:** |  | | **Nationality:** |  |  |
| **Number of Absentees:** |  | **Reasons:** |  | | | |  |
| **Are there any dropout?** |  | **Reasons:** |  | | | |  |
| **Session title:** | Looking back, Looking forward | | | | | |  |
| **Facilitator Name:** |  | | **Co-facilitator name:** | | |  |  |
|  | | | | | | | |
|  | | | **Yes** | **No** | **N/A** | **Comments** | |
| **1** | **Were all the resources ready for the session?** | |  |  |  |  | |
| **2** | **Did you welcome the participants?** | |  |  |  |  | |
| **3** | *Did you say the welcoming sentence as it is or in a very similar way?* | |  |  |  |  | |
| **4** | **Did you do Exercise 1 "Fast around the circle" or "Observation Rows"?** | |  |  |  |  | |
| **5** | **Did you check if they did the Home Practice?** | |  |  |  |  | |
| **6** | *Did you follow the exercise from manual as it is?* | |  |  |  |  | |
| **7** | *Did you say the connection point as it is or in a very similar way?* | |  |  |  |  | |
| **8** | **Did you do Exercise 2 "Review of main learning points"?** | |  |  |  |  | |
| **9** | *Did you follow the exercise from manual as it is?* | |  |  |  |  | |
| **10** | *Did you say the connection point as it is or in a very similar way?* | |  |  |  |  | |
| **11** | **Did you do Exercise 3 "Personal Reflections"?** | |  |  |  |  | |
| **12** | *Did you follow the exercise from manual as it is?* | |  |  |  |  | |
| **13** | *Did you say the connection point as it is or in a very similar way?* | |  |  |  |  | |
| **14** | **Did you do Exercise 4 "Positive Facilitator Feedback"?** | |  |  |  |  | |
| **15** | *Did you follow the exercise from manual as it is?* | |  |  |  |  | |
| **16** | *Did you say the connection point as it is or in a very similar way?* | |  |  |  |  | |
| **17** | **Did you assign the home practice?** | |  |  |  |  | |
| **18** | **Did you check-out?** | |  |  |  |  | |
|  | | | | | | | |
| **19** | **Were there any comments or concerns from the caregivers?** | | | | | | |
|  | | | | | | | |
|  |  |  |  |  |  |  |  |
|  |  |  |  |  |  |  |  |
|  |  |  |  |  |  |  |  |
| **20** | **Were there any comments or concerns from the facilitators?** | | | | | | |
|  | | | | | | | |
|  |  |  |  |  |  |  |  |
|  |  |  |  |  |  |  |  |
|  |  |  |  |  |  |  |  |

**Additional File 5. CSI Skills Use tool^^[[1]](#footnote-1)^^**

Introduction: People do different things to reduce their tension in their lives and in their family. Please let us know if you do any of the following activities.

1. In the past 2 months, how much have you used **specific techniques or behaviors to manage your stress, or to feel more relaxed** *(for example deep breathing, stepping back, peaceful walks)*?

0=Not at all

  1=A little/rarely (once or twice in the past month)

               2=Sometimes (about once a week)

               3=Most of the time (a few times per week)

               4=All the time (almost every day)

1. In the past 2 months, how much have you used **specific techniques or behaviors to control feelings of anger or frustration** *(for example taking a break, counting, taking the other perspective)*?

0=Not at all

  1=A little/rarely (once or twice in the past month)

               2=Sometimes (about once a week)

               3=Most of the time (a few times per week)

               4=All the time (almost every day)

1. In the past 2 months, how much were you able **to give positive attention to, and spend quality time with, your child(ren)**?

0=Not at all

  1=A little/rarely (once or twice in the past month)

               2=Sometimes (about once a week)

               3=Most of the time (a few times per week)

               4=All the time (almost every day)

1. In the past 2 months, how much **were you able to discipline your child(ren) without responding with yelling or hitting** *(for example giving praise, avoiding escalation)*?

0=Not at all

  1=A little/rarely (once or twice in the past month)

               2=Sometimes (about once a week)

               3=Most of the time (a few times per week)

               4=All the time (almost every day)

1. In the past 2 months, how much have you **gained knowledge about what is important for the healthy development and upbringing** of your child(ren)?

0=Not at all

1=A little

2=Moderate

3=Quite a lot

4=Very much

1. The tool is developed as a measure of the intervention mechanism of action. It measures

   the use of behavioral and psychosocial skills related to CSI content, aiming

   to evaluate skill acquisition relevant for CSI. Each of the intervention’s active ingredients

   (stress management through deep breathing, problem-solving, behavioral activation,

   and seeking social support) are represented in the instrument. A total score indicating

   the level of combined skill acquisition (score range = 0 to xx). For implementation purposes, it is developed to routine assess whether participation in the intervention results in the desired behavior change, which subsequently results in the changed outcomes. For research purposes, the instrument is developed to be used among both CSI and control-group participants, it is worded so that it is relevant for both groups. [↑](#footnote-ref-1)
